# Sediment Source Fingerprinting of the Lake Urmia Sand Dunes

**DOI:** 10.1038/s41598-017-18027-0

**Published:** 2018-01-09

**Authors:** Hesam Ahmady-Birgani, Edris Agahi, Seyed Javad Ahmadi, Mahdi Erfanian

**Affiliations:** 1 Faculty of Natural Resources, Urmia University, Urmia, Iran; 2 Nuclear Science and Technology Research Institute, Atomic Energy Organization of Iran, Tehran, Iran

## Abstract

Aeolian sand dunes are continuously being discovered in inner dry lands and coastal areas, most of which have been formed over the Last Glacial Maximum. Presently, due to some natural and anthropogenic implications on earth, newly-born sand dunes are quickly emerging. Lake Urmia, the world’s second largest permanent hypersaline lake, has started shrinking, vast lands comprising sand dunes over the western shore of the lake have appeared and one question has been playing on the minds of nearby dwellers: where are these sand dunes coming from, What there was not 15 years ago!! In the present study, the determination of the source of the Lake Urmia sand dunes in terms of the quantifying relative contribution of each upstream geomorphological/lithological unit has been performed using geochemical fingerprinting techniques. The findings demonstrate that the alluvial and the fluvial sediments of the western upstream catchment have been transported by water erosion and they accumulated in the lower reaches of the Kahriz River. Wind erosion, as a secondary agent, have carried the aeolian sand-sized sediments to the sand dune area. Hence, the Lake Urmia sand dunes have been originating from simultaneous and joint actions of alluvial, fluvial and aeolian processes.

## Introduction

Desertification processes, sand dune formation and wind-blown sand movement in arid and semi-arid areas are critical problems^1–6^. It has been shown that these desertification processes had taken place in different ecosystems and various environments based on both environmental and anthropogenic agents^1,7–10^. Today, the lake water level fluctuations are influenced by the consequences of high water consumption development plans, harsh agricultural activities, man-made changes to the system and upstream contest over water after prolonged drought^11,12^, thereby leading a large increase in desertification. The drying of lakes can have an impact on all environmental and ecological functions, including abiotic and biotic factors^13,14^ associated with socio-economic conflicts^15,16^ with saltwater intrusion^17–19^, groundwater salinization in coastal and lakeside aquifers^20–22^ and wind erosion and dust emissions^23–26^.

Sand dunes and sand seas containing aeolian deposits are geomorphological features^27,28^ that are distributed all over the world including Australia, Iran, the Middle East region, Northern Africa, the Arab States of the Persian Gulf and Sea of Makran coasts, where sand supply, topographic conditions and wind regime play key roles in sand accumulation^29–33^.

The origin of aeolian sediments and sedimentary environments, including loess deposits and historic sand seas, remains an unanswered question. In addition to the investigations of the early, mid and late Holocene sand seas and sand dunes^34–39^ and the loess deposits of the Late Miocene, Pliocene and last glacial periods^40–43^, over recent decades because of anthropogenic impacts and global climate change, newly-born sand dunes around dried lakes are being formed (for example: the Lake Urmia sand dunes, Northwestern Iran; the Owens Lake dune fields, California, USA). It is unclearly supposed that the materials are shedding off nearby upstream catchment. Therefore, a study on the sediment supply from possible sources seems necessary. Previous studies have targeted the chronology and the dating of sand dunes by using optically stimulated luminescence (OSL), infra-red stimulated luminescence (IRSL) and thermo-luminescence (TL) techniques, with high probably uncertainties in the results^44,45^. As the geologically uniformitarianism doctrine states, ‘the present is the key to the future’, having now evaluated geomorphological landforms and units being created can help us improve our understanding of geomorphological processes. Accordingly, the sediment fingerprinting technique is routinely used to identify the origin of sediments in a basin as an efficient method^46–53^. It relies on natural tracers that are the characteristics of the sediment at its source; they quantify the source of sink sediments from source sediments.

Recently, after desiccating Lake Urmia in Northwestern Iran, sand dune ridges and sand dunes similar to sand seas are being created as a new geomorphological landform. Without having dust and sand storms plus glacial processes over the region at present, the possibility of a relationship between the aeolian processes of the Lake Urmia sand dunes and the alluvial/fluvial processes of its western upstream catchment must be reasonably evaluated. This means that the role and the proportion of alluvial and fluvial processes on an aeolian environment of Lake Urmia as a research question of international significance should be more accurately quantified. Thus, identification of the dominant processes and sources generating the sediment within its catchment are vital. The sand dune formation shows greater association with mechanical weathering. The geomorphological/lithological and geological properties of the catchment, therefore, seem very significant. Thus, the aim of the present study was to locate the potential sediment sources feeding the Lake Urmia sand dunes and to let authorities know how take conservative operations and proper actions across the catchment to prevent the expansion of the Lake Urmia sand dunes.

## Results

### Optimal Fingerprints

The representative elements (i.e., Ag, Al, Ba, Ca, Cd, Co, Cr, Cu, Fe, K, Li, Mg, Mn, Na, Ni, Pb, Sr and Zn) were selected by the Kruskal-Wallis H-test to discriminate among the geomorphological/lithological units. The elements Ba, Ca, Cd, Co, Cu, K, Mn, Ni and Pb were eliminated on the basis of the fact that their *P*-values were above the 5% level of significance (i.e., P ≥ 0.05). Nine elements (Ag, Al, Cr, Fe, Li, Mg, Na, Sr and Zn) were below the 5% level of significance and were identified as representative fingerprint elements (Table 1).

**Table 1:** Descriptive statistics of each source sample tracer and P-values ≤ 0.05 from the Kruskal-Wallis H- test (^*^significant at P-values ≤ 0.05).

Tracers	N	Mean	Std. Deviation	Minimum	Maximum	Chi-Square	Asymp. Sig.
**Ag**	38	0.005850	0.0112148	0.0001	0.0660	22.992	0.002^*^
**Al**	38	52.646053	11.3648856	27.2300	72.5200	16.804	0.019^*^
**Ba**	38	0.123605	0.0820582	0.0130	0.3410	10.730	0.151
**Ca**	38	57.131842	44.7356544	14.0200	155.5000	6.286	0.507
**Cd**	38	0.001053	0.0007165	0.0001	0.0020	5.802	0.563
**Co**	38	0.022026	0.0061972	0.0100	0.0420	12.594	0.083
**Cr**	38	0.122816	0.0383496	0.0450	0.2620	15.683	0.028^*^
**Cu**	38	0.039974	0.0330703	0.0120	0.1680	13.012	0.072
**Fe**	38	37.085000	9.5045994	20.0600	59.8100	20.224	0.005^*^
**K**	38	16.656000	4.8153869	6.9790	25.8700	11.689	0.111
**Li**	38	0.021237	0.0077996	0.0120	0.0590	21.330	0.003^*^
**Mg**	38	17.908000	7.0310245	8.7580	47.8300	14.485	0.043^*^
**Mn**	38	0.728316	0.1850739	0.2760	1.1810	4.953	0.666
**Na**	38	12.458368	6.3041487	5.1250	35.6000	21.750	0.003^*^
**Ni**	38	0.060947	0.0193110	0.0230	0.1270	11.321	0.125
**Pb**	38	0.012079	0.0192738	0.0010	0.0970	10.050	0.186
**Sr**	38	0.209421	0.1747928	0.0910	0.8870	13.929	0.052^*^
**Zn**	38	0.170237	0.0346663	0.1250	0.2560	15.450	0.031^*^
**Source**	38	4.447368	2.0228074	1.0000	8.0000		

To optimise and eliminate the redundant tracers of the representative fingerprint elements (Ag, Al, Cr, Fe, Li, Mg, Na, Sr and Zn) that passed the Kruskal-Wallis H-test, the Stepwise Discriminant Function Analysis (DFA) was used. The optimum set of tracers (composite fingerprints) was gained by minimizing Wilks’ lambda (Table 2).

**Table 2:** The optimum set of tracers of DFA-induced outputs.

Variables Entered/Removed^a,b,c,d^													
Step	Tracers	Statistic	df1	df2	df3	Exact F				Approximate F			
						Statistic	df1	df2	Sig.	Statistic	df1	df2	Sig.
1	Ag	0.488	1	7	30	4.503	7	30	0.002				
2	Na	0.221	2	7	30	4.661	14	58	0				
3	Al	0.083	3	7	30					5.301	21	80.951	0
4	Zn	0.041	4	7	30					5.039	28	98.772	0
**Variables in the Analysis**													
**Step**					**Tolerance**			**F to Remove**			**Wilks’ Lambda**		
1	Ag				1			4.503					
2	Ag				0.858			5.505			0.516		
	Na				0.858			4.98			0.488		
3	Ag				0.794			5.941			0.207		
	Na				0.434			8.683			0.265		
	Al				0.503			6.618			0.221		
4	Ag				0.773			5.354			0.097		
	Na				0.41			9.08			0.137		
	Al				0.498			6.249			0.107		
	Zn				0.831			4.03			0.083		
**Classification Results** ^**a**,^													
**Original**	**Count**	**Source**	**Predicted Group Membership**								**Total**		
			**1**	**2**	**3**	**4**	**5**	**6**	**7**	**8**			
		1	3	0	0	1	0	0	0	0	4		
		2	0	3	0	0	1	0	0	0	4		
		3	0	0	3	1	0	0	0	0	4		
		4	0	1	0	4	0	1	0	0	6		
		5	0	1	2	1	2	1	0	0	7		
		6	0	0	1	2	0	4	0	0	7		
		7	0	0	0	0	0	0	4	0	4		
		8	0	0	0	0	0	0	0	2	2		
	%	1	75	0	0	25	0	0	0	0	100		
		2	0	75	0	0	25	0	0	0	100		
		3	0	0	75	25	0	0	0	0	100		
		4	0	16.7	0	66.7	0	16.7	0	0	100		
		5	0	14.3	28.6	14.3	28.6	14.3	0	0	100		
		6	0	0	14.3	28.6	0	57.1	0	0	100		
		7	0	0	0	0	0	0	100	0	100		
		8	0	0	0	0	0	0	0	100	100		

At each step, the variable that minimizes the overall Wilks’ Lambda is entered.

(**a**) Maximum number of steps is 18. (**b**) Minimum partial F to enter is 3.84. (**c**) Maximum partial F to remove is 2.71.

(a) 65.8% of original grouped cases correctly classified.

**Figure 1:**
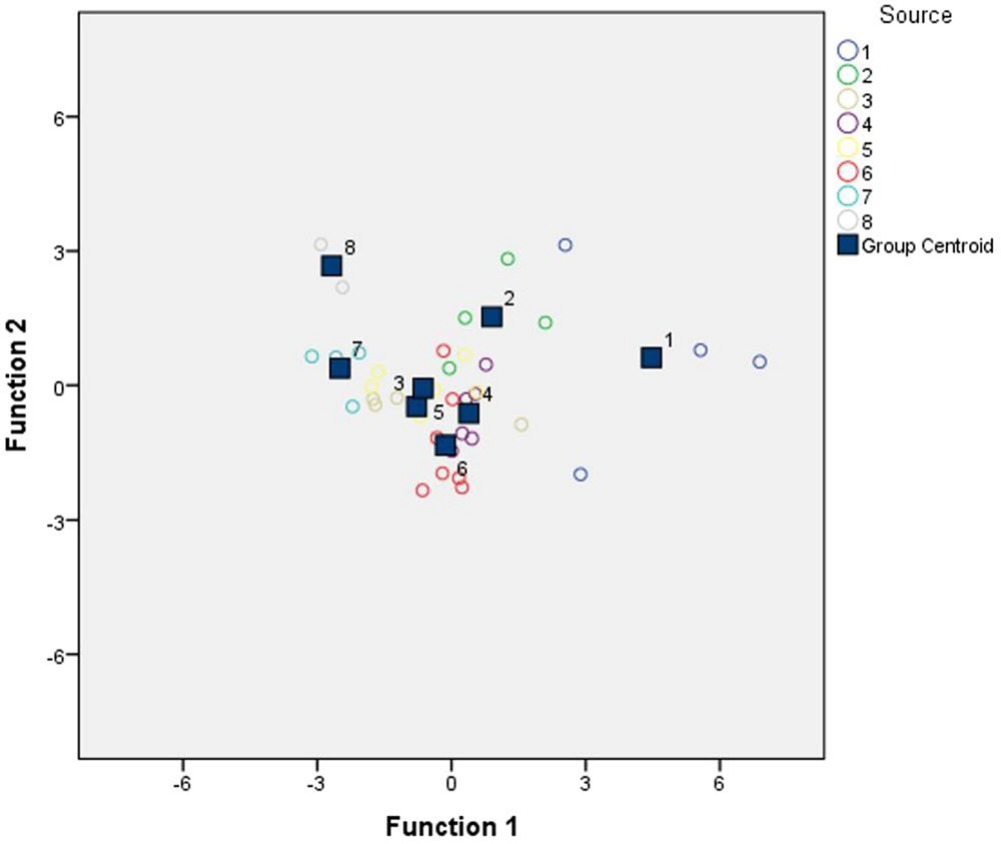
Canonical discriminant function of DFA outputs for lithological units by Ag, Na, Al and Zn elements. The farther the Group Centroids, the less errors of classification likely is. The Group Centroid overlap between the geomorphological/lithological units (No. 3 and 5) indicate the non-suitability of the traces used.

The minimum F to Enter value (3.84) and the maximum F to Remove (2.71) value showed the highest value for Ag (4.503), Na (4.661), Al (5.301) and Zn (5.039) elements. The mentioned tracers were inserted into the model at a given step. On the other hand, the lowest value for Cr (1.450), Fe (2.049), Li (0.669), Mg (1.711) and Sr (0.667) elements were observed and the tracers were removed from the model at a given step. On average, 66% of the sources were correctly classified, indicating that the selected elements (i.e. Ag, Na, Al and Zn) were rather strong discriminators among the sources (geomorphological/lithological units). With the *P*-value less than 0.05, it was also concluded that the corresponding function explained the group membership well. To test significance differences among groups, the Wilks’ lambda test was used. Smaller values of the Wilks’ lambda test represented the improved discriminatory capability of the model (i.e., Ag and Zn). The results showed that there were differences among the means of the various sources based on Wilks’ lambda test (Table 2). Nevertheless, the DFA results were visualised by the canonical discriminant function. The separation among the sources (geomorphological/lithological units) was rather pronounced; this was achieved by qualified fingerprints (i.e., Ag, Na, Al and Zn). The overlap areas among the sources (Source No. 3 and 5) were indicative of the non-suitability of certain traces in distinguishing the geomorphological/lithological units of the Kahriz catchment (Fig. 1).

### Discriminating Suppliers of the Lake Urmia Sand Dunes

**Figure 2:**
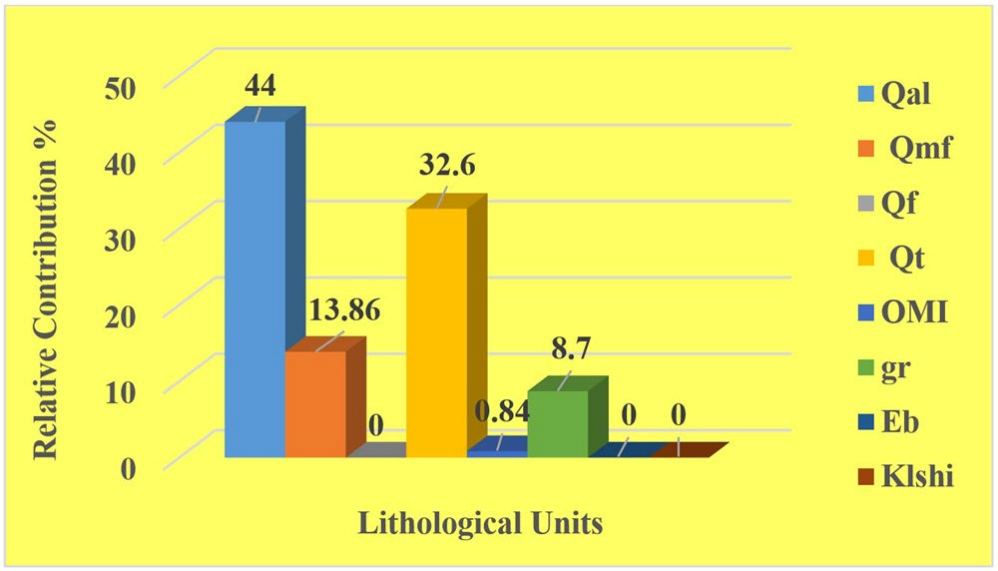
Relative contribution (%) for each geomorphological/lithological unit in the sediment source fingerprinting of the Lake Urmia sand dunes. As the graph shows, the recent channel deposits (Qal), alluvial fans and terraces (Qt) and sandy salty flats (Qmf) in the geomorphological units have the highest contribution in the formation of Lake Urmia sand dunes.

Identifying quantitative sources supplying the sand dunes across the western shore of Lake Urmia was achieved using statistical analysis and a mathematical procedure (multi-variate mixing model). The multi-variate mixing model was run in Microsoft Excel 2013 using the Solver command. The role of each geomorphological/lithological unit in the feeding of the sand dunes of Lake Urmia has been shown in Fig. 2. The results showed that the majority of the sand sediments were shedding off the recent channel deposits (Qal), alluvial fans and terraces (Qt), sandy salty flats (Qmf) and somehow the Rhyolite and marginal facies of granitic gabbro (gr) geomorphological/lithological units in descending order, with lesser proportions (<8%), even null of gravel fans (Qf), limestone, dolomitic limestone and silty shale (OMI), Cherty dolomite and metamorphosed sandstone (Eb) and Thin bedded shales with dolomitic limestone (KLsh1). Therefore, the relative contribution of each source was accurately estimated.

## Discussion

The western upstream catchment people of Lake Urmia are currently being challenged by an increase in the appearance of sand dunes. These sand dunes are presently burying the farmlands, orchards, old roads and irrigation channels, with an accelerated speed in moving to the Jabal Kandi and the Gol Tapeh villages as well as Urmia industrial Zone No. 3. Fifteen years ago, the region was covered by the shallow water of Lake Urmia; however, now the lake water has slowly retreated 7 km from the shore and the village dwellers never ceased to be amazed by newly-born sand dunes covering their region very quickly. Therefore, finding out where the sand dunes were originating was very important and vital. The geochemical fingerprinting method applied in this study was outstanding. In addition, it also helped efforts to control the alluvial, fluvial and aeolian sediment transport to be practically applied over the studied area. Elemental and geochemical analysis using ICP-AES proved to be an effective technique at differentiating sediment samples derived from different locations throughout the catchment.

**Figure 3:**
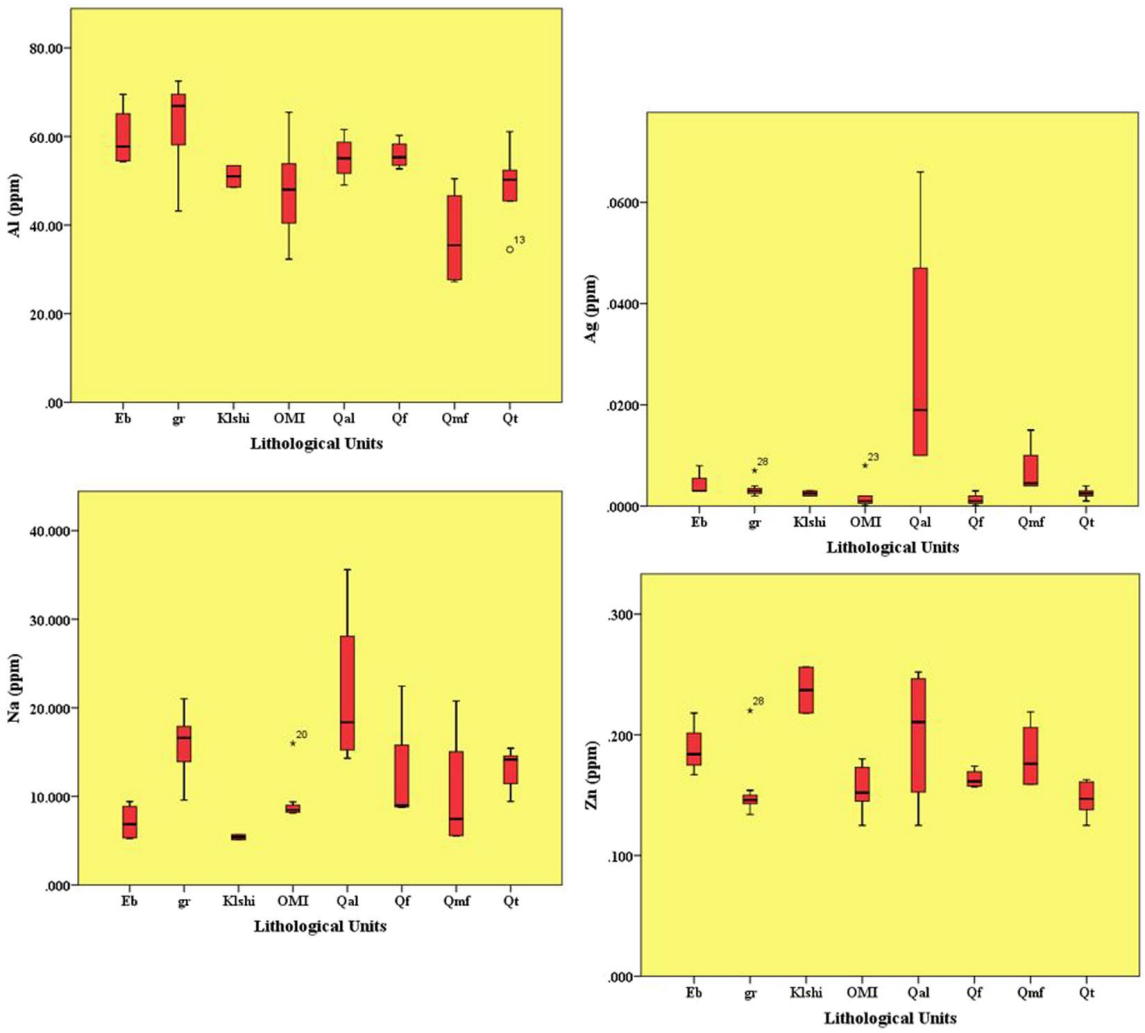
Mean concentration of Al, Ag, Na and Zn elements for each geomorphological/lithological unit is represented by DFA test. As the figure shows, for example, Al in the sandy salts flats (Qmf) is the highest in concentration and Ag, Na and Zn elements have the highest concentration in the recent channel deposits (Qal) among the other geomorphological/lithological units.

It is fully inferred that the clearest reason behind geochemical variability among the eight source areas was mineralogical and the geomorphological/lithological differences, which were associated with various land uses, land covers and the kind of water erosion. The findings showed that the mean concentrations of the four selected elements were presented by the DFA test (i.e., Al, Ag, Na and Zn). The separation of sources among the eight geomorphological/lithological units using these elements were to a large extent acceptable, though little overlap was observed. The element Al had the highest concentration (~37 ppm) in terms of the Qmf geomorphological unit; rest of the elements namely, Ag (~0.03 ppm), Zn (~0.2 ppm) and Na (~22 ppm) were higher in terms of the Qal geomorphological unit (Fig. 3).

Aluminium, as a major constituent of rocks and minerals, showed a high concentration in terms of the Qmf geomorphological unit, most likely due to the severity of soil erosion and the size-selective deposit transport within the catchment that help fine-sized particles specifically, clay minerals including kaolinite (Al_2_Si_2_O_5_(OH)_4_) and many rock-forming minerals (for example: feldspar, mica, amphibole, etc) deposit there, followed by low slope and the eastern aspect (Fig. 3). There is very little Al_2_O_3_ ranging from ~1–2.5% in carbonates, ~5–8% in sandstones and much higher concentrations ~15% in shale (because of the presence of clay minerals) for sedimentary rocks. The concentration of Al in igneous rock types commonly increase in parallel to a decrease in Fe and Mn contents. Mafic rocks, such as basalt, can contain up to 16% in terms of Al^54^. It could also be associated with anthropogenic sources such as sewage and atmospheric particulates^55^. Generally, an abundance of mica and clay minerals in deposit loads represent a relatively high Al content^54^. The accelerated weathering of aluminosilicates consists of mainly Al and Ag elements. The spatial variation of Al_2_O_3_ in the sandy salty flats (Qmf geomorphological unit) sediment was associated with the western upstream bedrock geology (granite, gabbro and shale) of the Eb, gr, OMI and Qmf geomorphological/lithological units; however, the distribution of clay-rich soil across the region is very effective as clearly seen in Fig. 3, upper left image (Al element).

The elements Ag, Na and Zn showed the highest concentration in the Qal geomorphological unit. Except for Na, the Ag and Zn elements form several minerals, including argentite (Ag_2_S), arsenargentite (Ag_3_As), sphalerite (ZnS), zincite (ZnO) and smithsonite (ZnCO_3_), however, they are widely dispersed as a trace element in pyroxene, amphibole, mica, garnet, magnetite, galena, sphalerite, tetrahedrite and chalcopyrite^56^. There is little Ag in calcareous rocks and elevated Zn represents mafic rocks. In sediment loads, Ag concentrations mostly vary from 0.05 to 0.12 mg kg^−1^ ^57^. If Zn is to be adsorbed by minerals and organic matters, it accumulates on the soil surface^58^. In sedimentary rocks, the abundance of ferromagnesian silicates and detrital oxides (for example: magnetite, and clay minerals) impacts the distribution of Zn^59^. Carbonate rocks (50 mg kg^−1^) and quartzo-feldspathic sand (30–50 mg kg^−1^) are generally depleted in Zn in comparison to greywacke (70–100 mg kg^−1^) and shale (50–90 mg kg^−1^). In the absence of Fe, Zn is adsorbed onto ferric oxides and is usually accompanied with silicate and carbonate phases. Zinc enrichment in sediment loads can be reasonably related to certain agricultural action pollutants such as liquid manure spreading.

Sodium-bearing minerals such as silicates (i.e., feldspar and Na-mica) are numerous. Except for ultramafic rocks, Na is a major component in all igneous rocks. Sedimentary rocks, such as limestone and dolomite, have the highest concentrations of Na (~5400 mg kg^−1^)^60^. For sedimentary rocks, detrital feldspar and clay minerals are the major sources of Na, and shale and sandstone rocks have Na concentrations of about 0.8 and 1.4% respectively^61^. High total Na_2_O in the floodplain sediment (>1.50%) occurs in areas with shale, feldspathic sandstone, felsic and intermediate rocks, such as granite, granodiorite and alkaline volcanic rocks^62^. Nevertheless, the source of potential soil contaminants includes pesticides and fertiliser uses, and Zn is the most likely element that is routinely used in farmland and orchards especially, in the Qt lithological unit. The findings of present study show that the Ag, Zn and Na elements were shed off the western upstream catchment into the recent channel deposits (Qal geomorphological unit) and the consistent sources of sediments were reasonably transported through the Kahriz catchment (i.e., Eb, OMI, Klshi and gr lithological units) (Fig. 3).

Overall, the use of the selected elements concentrations (i.e., Al, Ag, Zn and Na) were considered as suitable proxies for fingerprinting the sand dunes in the western shore of Lake Urmia and they were in full compliance with its upstream geological background. Therefore, there were no distant and out-of-the-region sediment sources arising from gale-force winds and feeding the sand dunes far-flung from the region.

Except for the Qt geomorphological/lithological unit (alluvial fans and terraces) covered by dense orchards and farmlands, the rest of geomorphological/lithological units (i.e., Eb, KLshl, OMI, Qal, Qf, Qmf and gr) have sparse and degraded vegetation cover because of livestock overgrazing and consequent damage to the thinly trampled soil and poor structure. However, the key role of land use in the formation of sand dunes beside the geomorphological/lithological units is not venial and it has strongly contributed to accelerating water and wind erosion in the region. Similar studies on the effect of land use and land cover in the sediment yield of upstream sources have been performed by others^46,63–66^. It has been demonstrated that soil erosion and deposition are not only functions of geomorphological/lithological units but they also vary as a function of factors such as the kind of land use and land cover in the regions.

**Figure 4:**
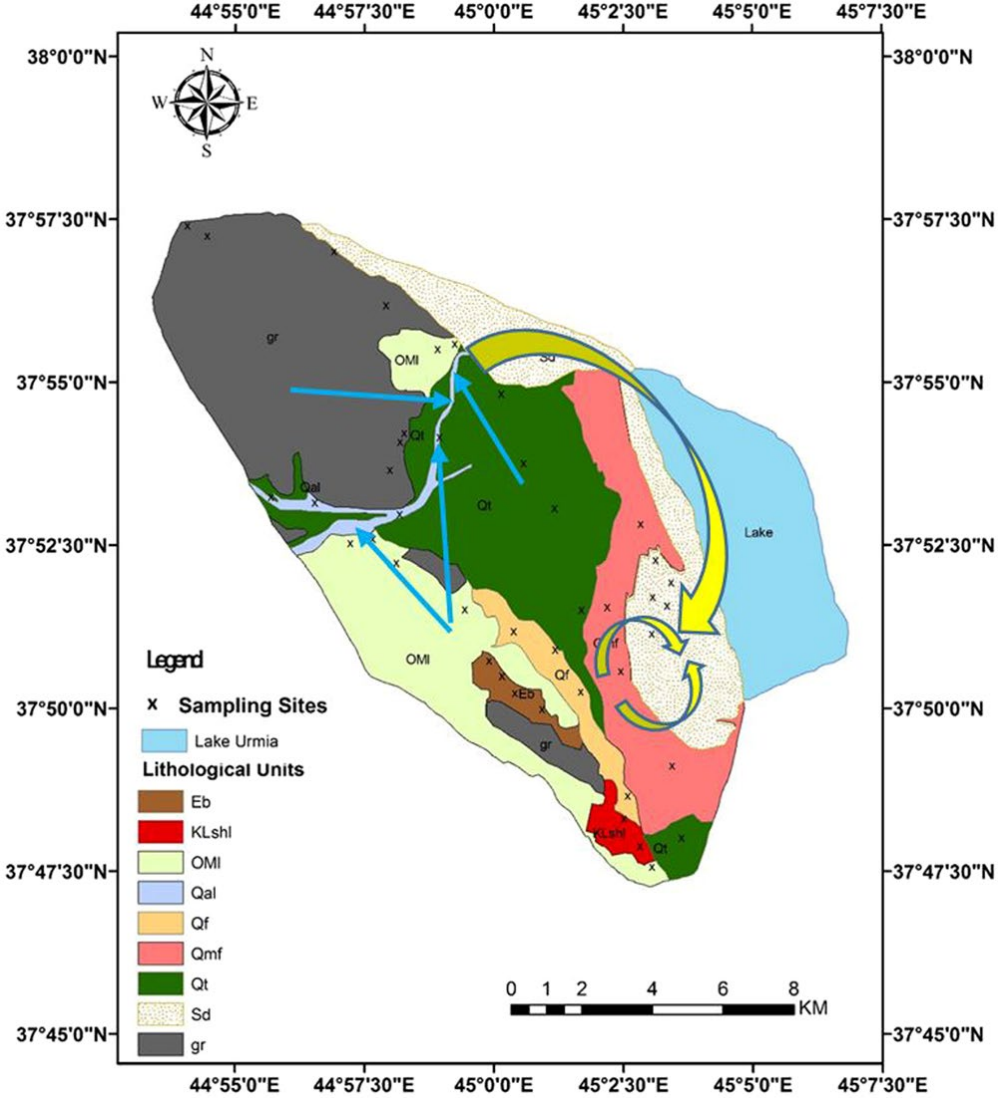
The blue arrows are representing the transportation of the sand-sized sediments by water erosion in upper parts of the catchment toward the Kahriz River as the primary sediment feeder and the yellow arrows are representing the role of wind erosion in formation of the sand dune as the dune shaper and secondary sediment feeder (Arc GIS software version 10.2).

As shown on the map, the Kahriz catchment is dominated by sedimentary and igneous rocks upstream with vast areas of quaternary deposits (alluvial fans and terraces) downstream, which surround the Kahriz River, which ends up in Lake Urmia. These sand dunes are derived from the upstream geomorphological/lithological and formation units by water erosion, which are transported to the lower reaches of the Kahriz River and get deposited there. Then, wind erosion within the dry seasons (i.e. July, August, September and October) lift the sediment deposited across the lower reaches of the Kahriz River and accumulate them in the southern part of the region, which is fully approved by the wind rose of the Urmia meteorological station. The fluvial and alluvial sediment loads, potentially present in the upper parts of the catchment, are carried by water during floods and are spread over the region. According to the prevailing wind, the fine-dried sediment materials form linear sand dunes in the western shore of Lake Urmia (Fig. 4). These results are in agreement with the findings of Hamdan *et al*.^67^ and Refaat and Hamdan^68^ on the aeolian dune sand of the Toshka area, Southeastern Western Desert, Egypt, and Lancaster *et al*.^69^ on the Owens lake dune fields, and Garzanti *et al*., 2013^69^ in the Arab states of the Persian Gulf.

The selected elements are present in the soil (source) and the sand dune (sink) samples are generally consistent with the upstream geomorphological/lithological and formation units, particularly Qt, OMI, Qal, Qmf and gr. These potential source areas are also supported by their areas. It should be noted that the smaller the areas, the lesser their importance or role in sediment yield- especially for the Eb, the Klshl and the Qf geomorphological/lithological units. However, the characteristics of the slope, the aspect, the soil texture, the number of drainage networks, in close proximity to the main stream (the Kahriz River) and sink sources (sand dunes), are significant issues. For example, in the Qf geomorphological unit (gravel fans), the lands covered by large-sized pebbles, cobbles and rather boulders, which have prevented soil erosion and degradation, resulting in a neutral proportion (relative contribution of 0%) of sediment yield in the region. Nevertheless, the type of land use and land cover associated with dense vegetation cover clearly influenced the causative agent of erosion that was obviously seen in the Qt geomorphological/lithological unit (alluvial fans and terraces had a relative contribution of 32.6%). In this geomorphological/lithological unit, the role of wind erosion owing to vast and dense orchards and farmlands is negligible; therefore, only water erosion is convincingly accepted. In the Qmf (sandy salty flat) geomorphological unit, the sabkha environment was made by a combination of mud, salt and sand along the shoreline of Lake Urmia, which was characterized by evaporite-carbonate deposits with some siliciclastics. Dried-out sabkha sediments are susceptible to wind erosion and are easily transported by a light wind that is observed in the region. Consequently, the sediments from the Qmf geomorphological unit are directly carried toward the Sd unit (sand dunes). Therefore, Qmf has a significantly direct effect (relative contribution of 13.86%) on the formation of the Lake Urmia sand dunes. The other main source areas, namely Qal (relative contribution of 44%), Qt (relative contribution of 32.6%), gr (relative contribution of 8.7%) and OMI (relative contribution of 0.84%) routinely feed the Kahriz River and sand-sized particles are shedding off the mentioned sediment sources by water erosion, without almost any striking wind erosion over the western upstream catchment (Fig. 4).

Generally, at the moment, acceptably is known about how the sand dunes were formed across the western shore of the Lake Urmia and where they are sourced. For newly-born sand dunes and sand seas made in the present geological period, looking at inner geomorphic processes, the topographic conditions and the erosional processes of each individual basin are very important and impossible to ignore. The interconnected relationship among the alluvial, fluvial and aeolian processes generating sediment loads have been changing landforms and making new geomorphological structures after the Earth’s crust started changing due to natural or anthropogenic agents. However, further sedimentological and mineralogical studies in Phase Two of the study will be dealt with. Combining field measurements analysis with remote sensing techniques is vital for understanding land degradation processes and the formation of sand dunes as emphasized by Ahmady-Birgani *et al*.^1^, Feng *et al*.^2^ Xue *et al*.^70^ and Li *et al*.^71^. The present research provided useful knowledge on the origin of the Lake Urmia sand dunes using the geochemical fingerprinting technique. The results provided more precise information on the hazard assessment of sand dune movement and the encroachment that could assist the authorities in making the better decisions on soil and water conservation proceedings.

## Materials and Methods

### Study Area

**Figure 5:**
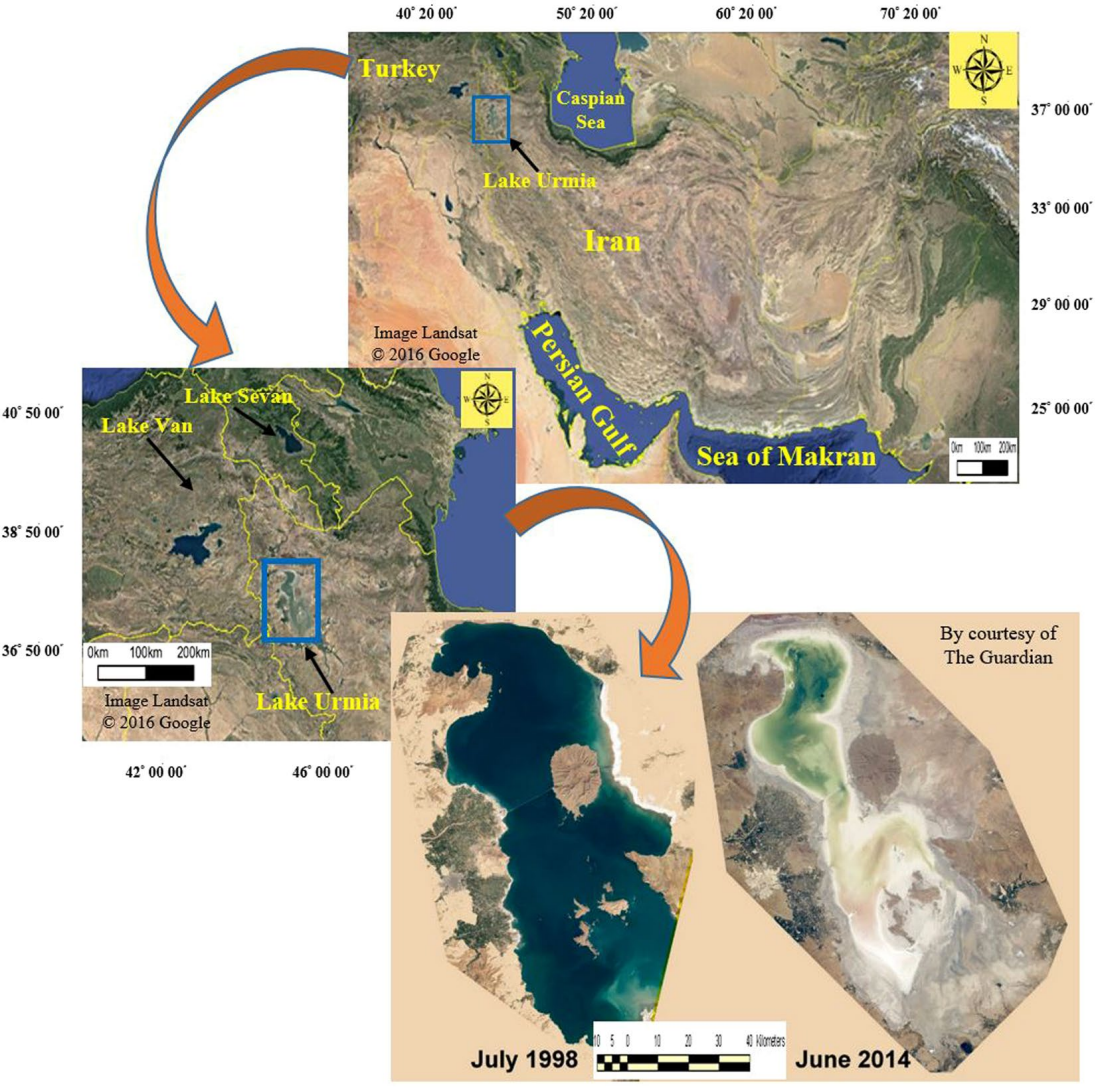
Location of the Lake Urmia, northwestern Iran (blue rectangle) is seen. As the figure shows, the Lake Urmia has lost more than 90 percent of its surface area over the past sixteen years. At the moment, the topographic conditions of Lake Urmia bed has changed from a bowl-shaped landform to a quite flat surface (Image from Google Earth Software).

Lake Urmia, the world’s second largest permanent hypersaline lake^72^ lies in Northwestrn Iran between latitude of 37.4°N and 38.15°N and longitude of 45.3°E and 46.2°E; its surface area diminished from 6000 km^2^ in 1972 to 1000 km^2^ presently (Fig. 5). The Increase in salt crusts, including sodium, chloride, sulphate, etc. in contents ranging from 14–34% through the Lake’s shoreline retreat are openly exposed to wind forces^73^ and damage the surrounding community of a population of around 6 million people with salt storms, they impact local agricultural lands and subsistence, and regional human health^16,74,75^.

The lithology of the Lake Urmia basin is diverse. There are metamorphic rocks to the west and coralline limestones to the northwestern shores, and dolomite, sandstone, quartzite and volcanic rocks in the south. Throughout the eastern islands and the eastern shore, flysch rocks can be observed. The northern and the northeastern rocks comprise evaporite-marl and an upper red-bed conglomerate series^76^.

**Figure 6:**
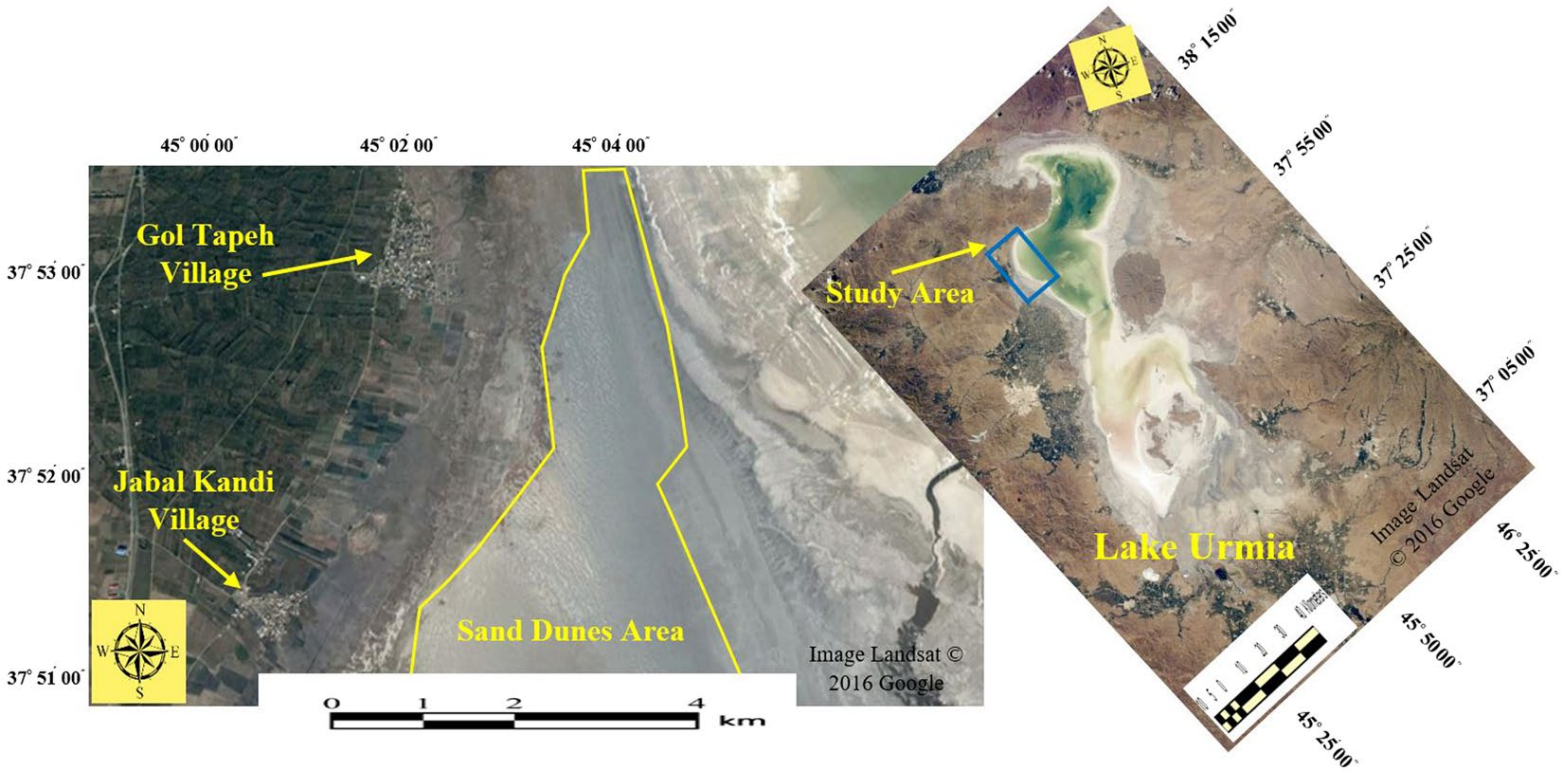
Location of the sand dune fields, the western shore of Lake Urmia. As the image shows, sand dunes are engulfing the surrounding agricultural lands and villages very fast. These sand dunes covering an area approximately 2000 ha, what were not fifteen years ago (Image from Google Earth Software).

Over the last decade, as soon as the Lake Urmia started drying up, sand dune fields were appeared through the western shores of the Lake’s bed retreat, covering an area of ~2000 ha and a perimeter of ~17.5 km along the Gol Tapeh village, the Jabal Kandi village and Lake Urmia (Fig. 6).

**Figure 7:**
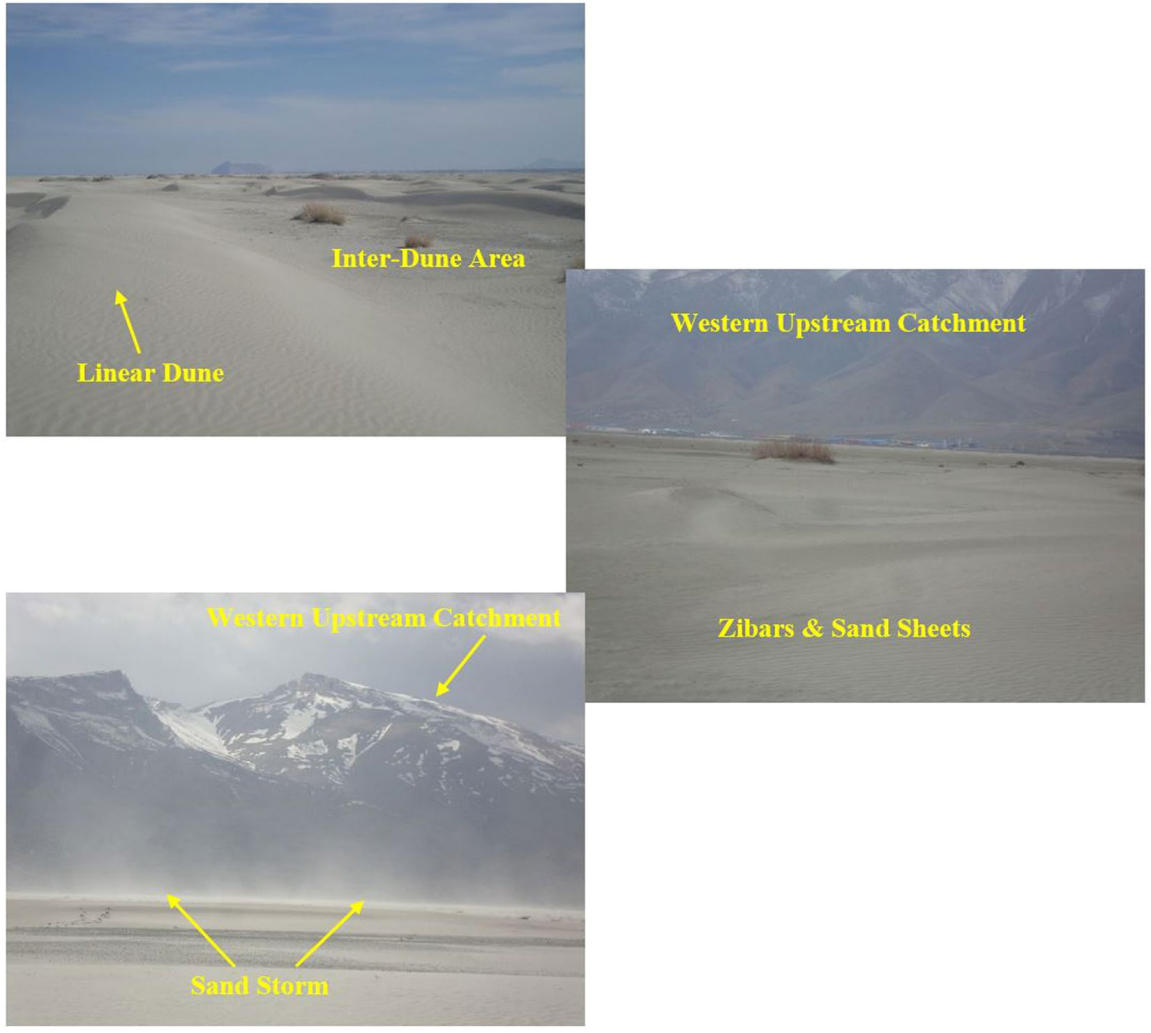
Areas of sand dune fields, the western shore of Lake Urmia and its surrounding catchment. The most frequent aeolian geomorphological landforms in the region are sand sheets, nebkhas, linear and barchan dunes. With erosive winds in the region, sand and dust storm is routinely seen.

The study area is bounded to the west by mountains; the most aeolian landforms, including sand sheets, nebkhas, linear and barchan dunes are present downstream (Fig. 7). These two types of sand dunes (linear and barchan) are placed as hyper active sand dunes and shift at highly varying rates and in various orientations^1^. The Kahriz River drains the western upstream catchment and ends up in Lake Urmia.

### Soil/Sediment Samples Collection

**Figure 8:**
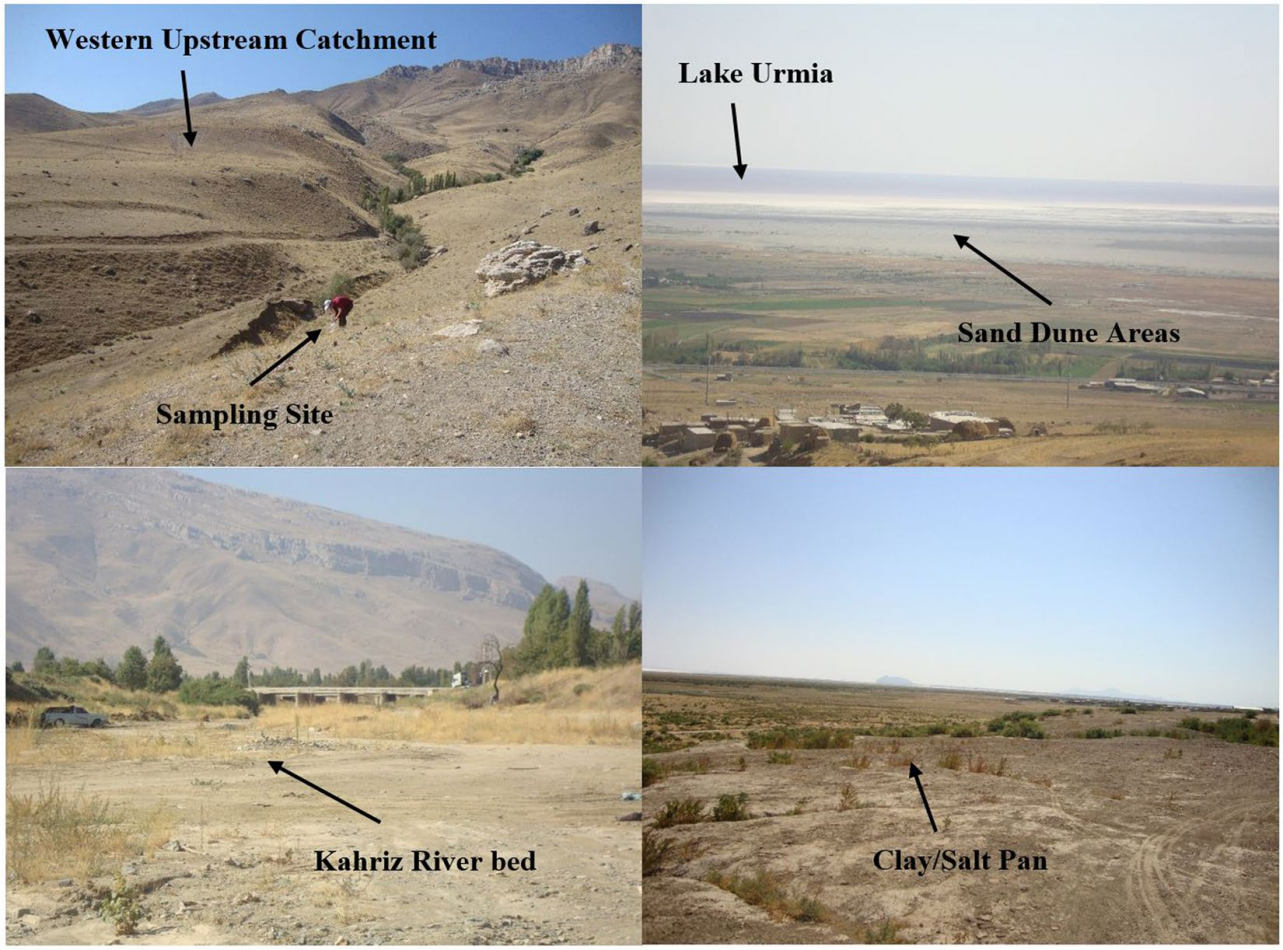
Potentially sediment sources, the western upstream catchment of the Lake Urmia. Locating the potential sediment sources feeding the Lake Urmia sand dunes is vital and the main aim of this study. With sediment source fingerprinting techniques, the aim of this study can be precisely done.

During fieldwork, 43 representative topsoil samples were taken from the source areas (geomorphological/lithological units) as the ‘sediment sources’ and deposition areas (sand dunes) as the ‘sink sources’. Geological or geomorphological/lithological sources were delineated as spatial sources^51,77–80^. Certain factors during sampling, such as topography and erosional processes, were also considered to combine the spatial source and the erosional processes^81,82^. It helped to directly identify the geomorphological/geological processes responsible for sediment generation (i.e., soil samples were grabbed within the upper 2 cm of the soil layer in the undisturbed zone, uncultivated fields, hillslopes overlooking the tributaries and streams, and in the proximity of major types of soil erosion such as sheet, rill, gully or bank erosion) (Fig. 8).

**Figure 9:**
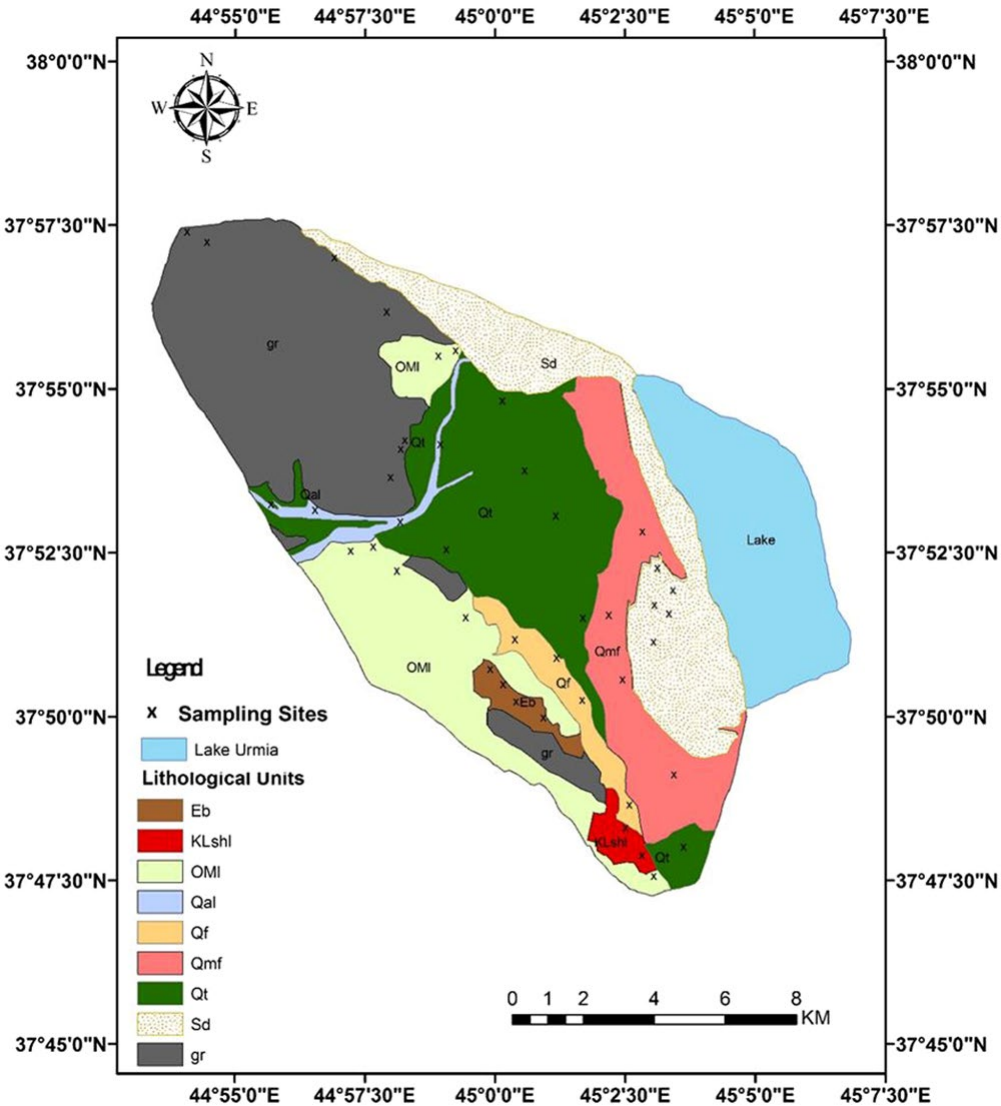
Potentially sediment sources (geomorphological/lithological units), the western upstream catchment of Lake Urmia. The crosses throughout the map show the location of the soil and sediment sampling sites. Appropriate distribution of sampling sites throughout the geomorphological/lithological units of the study area help to decrease the uncertainties of the results (Arc GIS software version 10.2).

For each geomorphological/lithological unit soil sample described in Table 3, approximately 45 sub-samples were considered along a transect, they were combined and produced a single composite sample (Fig. 9). This helped consider small-scale random variability in source material properties and limit the number of samples that require analysis to determine their properties^46,48,66^.

**Table 3:** Description of each lithological and geomorphological unit in the western upstream catchment of the Lake Urmia.

lithological and geomorphological Units	Description
Eb	Cherty dolomite, sandy argillitic shales & metamorphosed sandstone
KLshl	Thin bedded shales with thick bedded orbitoline limestone & dolomitic limestone
OMI	Reefal limestone, dolomitic limestone & cherty dolomitic & silty shale
Qal	Recent channel deposits
Qf	Gravel fans
Qmf	Sandy salty flats
Qt	Alluvial fans and terraces
Sd	Sand dunes
gr	Rhyolite and marginal facies of granitic batholiths, slightly mylonitized, Agmatite, Layered gabbro, Massive gabbro, norite, Dunite

**Figure 10:**
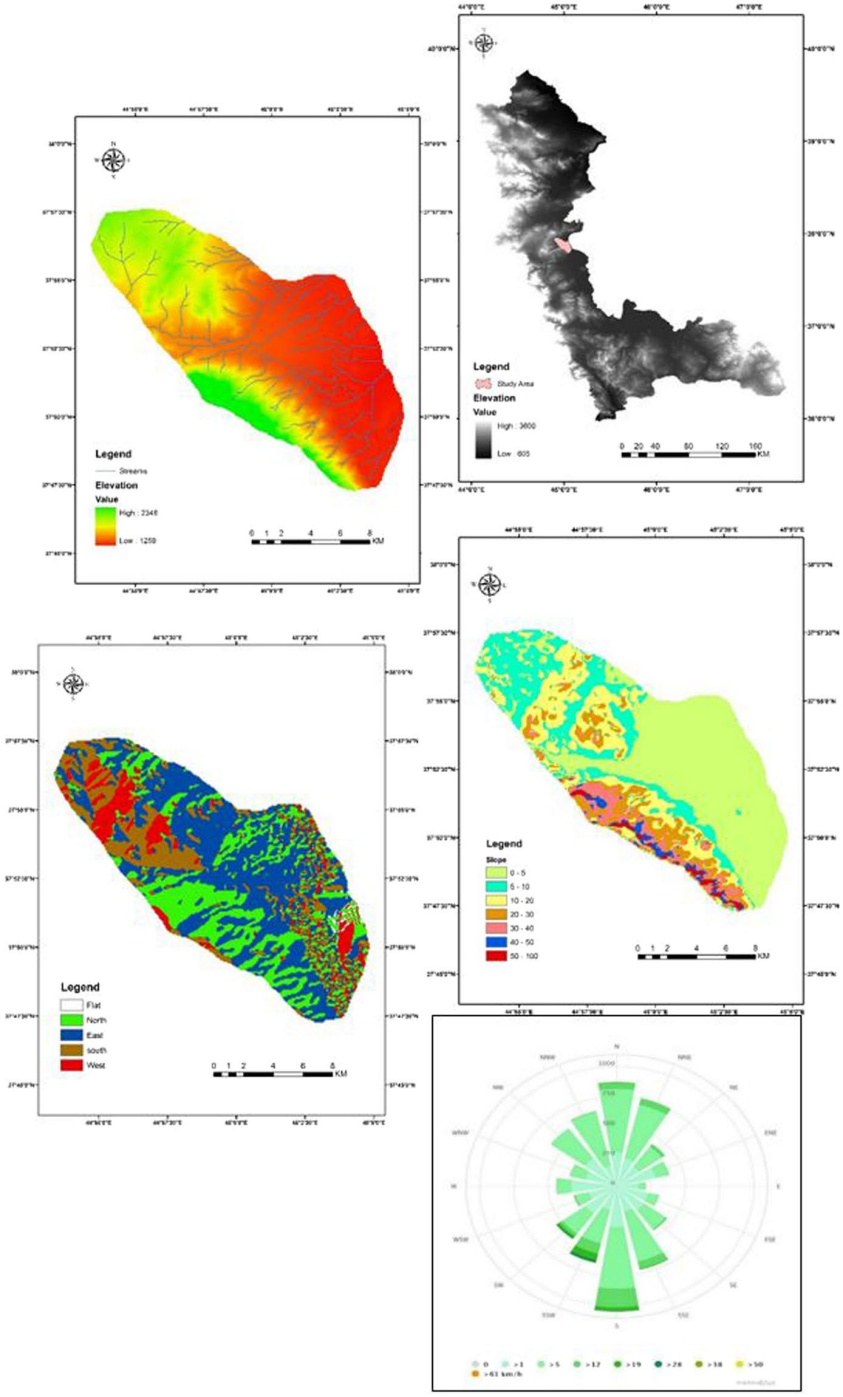
Location of the Kahriz catchment in the western upstream catchment of Lake Urmia. The drainage networks slope and aspect maps as well as wind rose of the study area is seen (Arc GIS software version 10.2).

The western upstream catchment (the Kahriz catchment) is an open catchment that covers an area of 164 km^2^, with a cold semi-arid climate (Köppen climate classification), a mean annual precipitation of ~350 mm and slope gradient and elevation range from flat to 75° and 1259–2346 m, respectively. At the Urmia meteorological station, wind directions cluster in the N and NNE (0–25°) and S, SSW and SW (170–225°), with maximum wind speeds ranging from 5–28 km.h^−1^. The most erosive winds for the western shore of Lake Urmia is from the southern and the south-western directions, with lower speeds from the east and the west (Fig. 10). According to the Kahriz catchment area (164 km^2^), the usage of geochemical-based fingerprinting methods to small catchments (<500 km^2^) is preferable for its ease and accuracy^83^.

### Geochemical Analysis of Soil/Sediment Samples

The samples grabbed from the watershed were sealed up; any sample properties were recorded (i.e., the type of geomorphological/lithological unit, coordinate points, elevation, land use and distance to the stream or river) and then transferred to the Central Laboratory, Faculty of Natural Resources, Urmia University. A number of samples were oven dried at 30 °C and some samples were exposed to open-air drying by a pestle and mortar disaggregated manually, if needed. Two kilograms of each sample collected was dry sieved using a shaker machine. Then, the fine fractions of <63 µm (mesh number = 270) as coarse silt-sized and smaller particles were chosen^46–53,66^. Fractions <63 μm fractions were used because they constitutes over 95% of the sediment loads being transported in rivers in the form of suspension^84^ and over 65% during wind erosion process by mode of suspension and saltation soil movements^85^. Moreover, the chemically reactive characteristics of these fractions help trace elements to be adsorbed on silt and clay-sized particles and minerals^23^.

Generally, in order to determine the association between the source and the sink areas in erosion studies has been a major goal of researchers. Hence, sediment tracers as a technique to complete the shortcomings of traditional erosion or deposition processes measurements have been increased due to the complementary information they make. The application and soundness of sediment fingerprinting techniques using a broad range of materials and soil properties such as major and minor elements as well as particles mineralogy^49,50,66,86,87^, fallout radionuclides^48,82,88–91^, rare earth elements and soil magnetism^66,92–95^ and organic and biological materials plus biomarkers^46,96–98^ have been evaluated in numerous studies^99^. In the present study, the sample preparation and geochemical analysis of major and trace elements were performed at the Atomic Energy Organization of Iran. For the objectives of this study, we selected the United States Environmental Protection Agency (USEPA) Digestion Method No. 3052 (mixed HNO_3_+HCl+HF)^100^ with microwave-digestion, which is now widely applied in environmental geochemical studies of soils, sediments and dust if a total decomposition analysis is required. Half a gram of each sample collected was digested in 9 mL of concentrated HNO_3_ and 3 mL of HF for 15 minutes using a suitable microwave heating system. HCL acid and H_2_O_2_ reagent were used as additional alternative acids. The samples volume was scaled up to a maximum of 1.0 g in inert polymeric microwave vessels. The vessels were sealed and heated in a microwave set at 180 ± 5 °C for nearly six minutes and around 10 minutes, respectively, for the completion of chemical reactions at 180 ± 5 °C. An amount of 2 ± 2 mL of concentrated HCL added to HNO_3_ and HF acids for stabilizing Ba, Sb, and Ag and elevated concentrations of Al and Fe in solution. The digests were quantitatively delivered to centrifuge tubes and diluted with ultrapure water to 30 ml. The tubes were then centrifuged and 1 ml of the digests were transferred into a 10 ml centrifuge tube and diluted to 10 ml with inductively coupled plasma- atomic emission spectroscopy (ICP-AES) internal standard.

The solutions of 18 elemental fingerprints were geochemically analysed for Ag, Al, Ba, Ca, Cd, Co, Cr, Cu, Fe, K, Li, Mg, Mn, Na, Ni, Pb, Sr and Zn by ICP-AES. Calibration was performed using a complex external standard (AccuStandards 1–5), covering the full mass range, with a correction for the blank inclusion of blank acid solutions. The Montana Soil Standard Reference Material (MSRM 2710) was used as an external cross-checking reference for calibration and the assessment of the accuracy of extraction efficiency. The estimated standard errors for the solution analyses were less than 5%. The detection limits in the solution for the analysed elements were better than 0.1 ug/l.

### Statistical Analysis and Mathematical Procedure to Sediment Fingerprinting

For sediment fingerprinting, elemental tracers (i.e., major elements, trace elements and REE_s_) are widely used as fingerprints in which soil erosion and sediment sources over a longer period are convincingly measurable. Therefore, the selection of the best tracers representing the source area are very important issue, which is made on the basis of the results of geochemical analysis that is being performed in laboratories.

Non-parametric tests were applied to compare the different fingerprints (tracers) of the different source areas. Non-parametric tests are free from distribution and make no hypothesis of the normal distribution of the population^101,102^. Two stages of statistical analysis were normally applied to distinguish between the sediments of the various sources (geomorphological/lithological units). At first, the Kruskal-Wallis H-test was used to retain those elements (P-value < 0.05) that could discriminate between eight sources (geomorphological/lithological units) and helped non-conservative elements to be eliminated. The remaining elements were introduced in the stepwise Discriminant Function Analysis (DFA) as a data reduction technique, which was helpful in determining whether a set of fingerprints were efficient in predicting source membership. Overall, an optimal source fingerprint is identified by DFA. The minimum number of fingerprint characteristics gains the greatest discrimination between the sediment sources according to the minimization of Wilks’ lambda. All the statistical analyses were carried out using the IBM SPSS Statistics Software, Version 23.

Finally, the outputs of the statistical analysis were transferred to the multi-variate mixing model and the relative proportion of each sediment source area (eight geomorphological/lithological units) were estimated by minimizing the Objective Function output (OF) as follows. The multi-variate mixing model and certain modifications of the correction factors are observable in Equation 1 ^103^.

1 $$OF=\sum _{i=1}^{n}={(\frac{{S}_{Sink}-({\sum }_{j=1}^{m}{S}_{Source}.{P}_{s}.{Z}_{s}.{O}_{s})}{{S}_{Sink}})}^{2}{W}_{i}\,0\le {P}_{S}\le 1\,\sum _{j=1}^{m}{P}_{S}=1$$

where:

OF: is the sum of squares of the residuals (Objective Function)

n: is the number of elements in the composite fingerprint

m: is the number of source groups (i.e. geomorphological/lithological units)

S_Sink_: is the concentration of the element property (j) in the sediment sink sample

S_Source_: is the mean concentration of the element (j) in source group (i)

P_S_: is the relative contribution/relative proportion from the source group (i) in the sediment sink sample (the goal of this research)

Z_S_: is the correction factor of particle size

O_S_: is the correction factor of organic matter

W_i_: is the tracer discriminatory weight.

For each of the composite fingerprint member elements, a linear equation was generated that related the mean concentration of the element in each source sample to the mean concentration in the sink sample. Hence, the composite fingerprint was presented by a set of linear equations- one for each element. The least squares method was used and the proportion from the different source areas/groups was estimated^77,104^.

## Conclusions

- Systematic and convincing variability in the relative proportion of the eight sources feeding the Lake Urmia sand dunes provided strong discrimination among the sediment sources (geomorphological/lithological units).
- The compositional and geomorphological/lithological diversity of the Kahriz catchment helped to distinguish sediment sources accurately by employing geochemical fingerprinting techniques.
- The Lake Urmia sand dunes originated from the alluvial and the fluvial sediments shedding off the western upstream catchment to the lower reaches of the Kahriz River, being transported by wind erosion to the sand dune fields.
- The aeolian sand dunes of the western shore of Lake Urmia loom over its neighbouring villages and industrial zones. The findings of the present study provided the locations of where soil and water conservation proceedings should be implemented.
